# Identification of radiographic characteristics associated with pain in hallux valgus patients: A preliminary machine learning study

**DOI:** 10.3389/fpubh.2022.943026

**Published:** 2022-08-10

**Authors:** ChenGuang Wang, Chao Li, Rui Zhang, ZhiJun Li, HuaFeng Zhang, Yuan Zhang, Shen Liu, XiaoYue Chi, Rui Zhao

**Affiliations:** ^1^Department of Orthopedics Surgery, Tianjin Medical University General Hospital, Tianjin, China; ^2^Department of Orthopedics, Xiangyang Central Hospital, Affiliated Hospital of Hubei University of Arts and Science, Xiangyang, China

**Keywords:** hallux valgus, multi-variate pattern analysis, pain, support vector machine, hierarchical clustering

## Abstract

**Objective:**

To investigate the association between the structural deformity and foot pain in hallux valgus (HV) patients using a multi-variate pattern analysis (MVPA) approach.

**Methods:**

Plain radiographic metrics were calculated from 36 painful and 36 pain-free HV feet. In analysis 1, univariate analyses were performed to investigate the clinical and radiographic differences between painful and pain-free HV. In analysis 2, we investigated the pattern differences for radiographic metrics between these two groups using a MVPA approach utilizing a support vector machine. In analysis 3, sequential backward selection and exhaustive search were performed as a feature-selection procedure to identify an optimal feature subtype. In analysis 4, hierarchical clustering analysis was used to identify the optimal radiographic HV subtype associated with pain in HV.

**Results:**

We found that: (1) relative to feet with pain-free HV, the painful ones exhibited a higher hallux valgus angle, i.e., the magnitude of distal metatarsal and phalangeal deviation; (2) painful HV could be accurately differentiated from pain-free HV *via* MVPA. Using sequential backward selection and exhaustive search, a 5-feature subset was identified with optimal performance for classifying HV as either painful or pain-free; and (3) by applying hierarchical clustering analysis, a radiographic subtype with an 80% pain incidence was identified.

**Conclusion:**

The pain in HV is multifactorial and associated with a radiographic pattern measured by various angles on plain radiographs. The combination of hallux valgus angle, inter-phalangeal angle, distal metatarsal articular angle, metatarsal cuneiform angle and metatarsal protrusion distance showed the optimal classification performance between painful and pain-free HV.

## Introduction

Hallux valgus (HV) is characterized as a combined deformity with mal-positioning of the first metatarsophalangeal joint caused by a lateral deviation of the great toe and a medial deviation of the first metatarsal bone. This can progress to significant metatarsophalangeal (MTP) joint subluxation, which is frequently encountered in foot and ankle clinic with a prevalence exceeding 20% in adults over 50 years of age (1). HV is often associated with the pain (2), impaired physical function (3) with consequent poorer general health (4) and diminished quality of life (5). Pain is the primary complaint related to HV (6), and typically the severity of the deformity directly correlates with the degree of pain. A prior publication noted a positive correlation between self-reported severity of HV (Short Form 36 or Foot Pain and Disability Index) and increasing levels of pain (5). However, there have also been conflicting reports of only a modest correlation between pain and structural HV deformity (i.e., HV angle measured from a radiographic image). Moreover, there have been contradictory reports concerning the link between foot pain and HV (5, 7–9). Discrepancies have been noted such as patients with a large HV angle yet without any pain, and others with only a mild HV angle that experience severe foot pain. The precise mechanism behind such phenomena has yet to be identified. In recent years, some authors have postulated alternative explanations that HV has affected the entire foot rather than just the MTP joint (10–12). When pain could not be explained by the degree of the HV angle alone, which has been traditionally used to evaluate the deformity of MTP joint, other radiographic angles, such as the intermetatarsal angle, and the metatarsal cuneiform angle have been implicated as potentially associated with pain in HV patients.

In recent decades, with the development of artificial intelligence and machine learning algorithms, multi-variate pattern analysis (MVPA) has been widely applied in the medical imaging field (13–15). MVPA methods provide a rich characterization of imaging data, often in a data-driven manner. This technique has enabled researchers to identify the key, significantly important parameters of a complex disease in a multi-variate manner rather than exploring univariate differences between the disease and healthy controls (15). Prior studies exploring the correlates of foot pain in HV patients have relied only on univariate analyses to determine the association between pain and HV deformity (e.g., two sample *T*-test or univariate correlation analyses between radiographic findings and degree of pain). Although the results of univariate analysis were straight-forward and easy to interpret, only linear-relations between the radiographic findings and clinical assessments were considered. Pattern information consisting of different radiographic metrics to evaluate the deformity of the entire foot was largely overlooked. More importantly, HV affected the structure of the entire foot rather than just the MTP joint (10–12). It has been shown that hallux valgus could affect the transverse arch structure and its force loading patterns (12). Furthermore, HV not only alters the geometry structure of forefoot but also causes biomechanics function changes of the foot (16, 17). A more comprehensive understanding of the deformity pattern associated with pain in HV would likely provide the basis for foot surgeons to develop novel surgical techniques to correct the HV deformity and resolve the pain.

Therefore, in this study, we first (1) conducted a MVPA to test whether the various radiographic angles could be used to classify painful HV patients from pain-free HV patients in a supervised manner; (2) performed a sequential backward selection and exhaustive search as a feature selection procedure to identify the optimal feature subset of radiographic features; and (3) carried out hierarchical clustering in an unsupervised manner to identify the radiographic HV subtype which was associated with pain in HV patients.

## Materials and methods

### Subjects

Written informed consent was obtained from each participant prior to each procedure. Ethical approval was granted by the Local Institutional Ethics Committee. The study comprised a total of 103 feet with HV, from 81 patients, recruited from January 2021 to January 2022 at both inpatient and outpatient departments of foot and ankle surgery. To control the degree of overall HV deformity, we used a HV angle above 20° as a cut-off value for patient recruitment rather than a traditional criterion for HV angle of >15° determined by standard radiographic images.

The exclusion criteria were as following: (1) history of foot or ankle surgery or trauma; (2) history of neurological diseases; (3) ankle joint or foot sprain within 3 months; (4) presence of concomitant hallux limitus (i.e., a minimum of 50 degrees of passive dorsiflexion at the first MTP joint was required) (2).

### Clinical assessment

The demographic data of the participants including sex, age, education years, ethnicity was obtained by questionnaire. The total years of education was determined from the patient's self-report of completed years of education. The Visual Analog Scale (VAS) was implemented to assess the degree of foot pain in each HV foot. Feet with a VAS score of 0 were identified as pain-free. Feet with VAS scores >3 extending more than 3 months were classified as painful (18, 19). Painless feet were selected from the dataset and excluded those that meet the exclusion criteria. Therefore, 36 painless HV feet were obtained. To further performed machine learning analyses and to avoid class-imbalance problem, 36 painful HV feet from the dataset to match the painless HV feet with age, gender, education years and ethnicity. Finally, 72 HV feet from 63 patients, 36 painful and 36 pain-free, were included in our study.

### Radiographic assessment

Bilateral weight bearing radiographs were obtained for all participants using a standardized procedure (tube to film distance 100 cm, angled 15° from vertical). All radiographic assessments were carried out in ImageJ toolbox. The assessment procedure of radiographic metrics we used were as follows. (1) Hallux Valgus angle (HVA): the hallux valgus angle is formed by the longitudinal axis of the first proximal phalanx and the longitudinal axis of the first metatarsus (20, 21). (2) Inter-Metatarsal Angle (IMA): the IMA, or metatarsus primus adducts angle, is the angle between the longitudinal axes of the first and second metatarsals (22). (3) Inter-Phalangeal Angle (IPA): the IPA is the angle between longitudinal axes of the proximal and distal phalanges (22). (4) Distal Metatarsal Articular Angle (DMAA): the DMAA is the angle between the perpendicular to the effective articular surface of the first metatarsal head and the longitudinal axis of the first metatarsal bone (22). (5) Distal Articular Set Angle (DASA): the DASA is the angle between the perpendicular to the effective articular surface of the 1st proximal phalanx and the longitudinal axis of the 1st proximal phalanx (22, 23). (6) Metatarsal Cuneiform Angle (MCA): the MCA is the measured angle between the longitudinal axis of the first metatarsal bone and the longitudinal axis of the medial cuneiform bone. (7) Metatarsal Adducent Angle (MAA): we used the method devised by Engel et al. (24) to measure the MAA. We measured the angle between the axis of the second metatarsal and the axis perpendicular to the transverse axis of the tarsus using the most lateral and most distal points of the joints of the cuboid, with the fifth metatarsal as a reference. (8) Metatarsal Protrusion Distance (MPD): located at the most distal extent of the second metatarsal bone, perpendicular to the longitudinal axis, this line reflects the distal-most protrusion of the second metatarsal. A line parallel to this is now constructed coursing through the distal-most point of the first metatarsal. The difference between these 2 parallel lines is the MPD (25). (9) Sesamoid Position (SP): The position of the tibial sesamoid was graded I, II, III, IV, V, VI, or VII according to its position relative to the functional longitudinal axis of the first metatarsal (26).

### Analysis 1: Univariate analyses for exploring radiographic differences between painful and pain-free HV feet

Two sample *t-*tests were performed for continuous variables and chi-square test was used for categorical variables to compare the baseline demographic data between two groups. Subsequently, Shapro-Wilk tests were performed to check the normal distribution of the radiographic measurements and QQ plots were also generated for illustrating normality of our data. All statistical analyses and figures were generated using GraphPad prism 9. Two sample *t-*tests were performed for continuous variables and chi-square test was used for categorical variables to compare the radiographic metrics between groups. Moreover, parametric test was performed for between group comparison for radiographic metrics which passed the Shapiro-Wilk test, otherwise nonparametric test was performed (i.e., Mann-Whitney test). The significant level was set to *P* < 0.05. Moreover, to elucidate the associations among clinical measures. Pearson correlation analyses were performed to demonstrate the association among radiographic metrics.

### Analysis 2: Multi-variate pattern analyses (MVPA) using a support vector machine (SVM) for classifying painful HV feet from pain-free ones

The SVM was use to distinguish painful HV feet from pain-free ones using radiographic features according to the homemade MATLAB script based on LibSVM's (http://www.csie.ntu.edu.tw/~cjlin/libsvm/) implementation of linear SVM using the default parameters: We used linear kernel, the penalty coefficient (*c*) was set to 1, other parameters were not adjustable therefore we made no further adjustment. To overcome the loss of generalization due to the relative sample size, the Leave-one-out-cross-validation (LOOCV) technique was used in this study. One of the available data-points in LOOCV was held-out, and the model was trained using the remainder of the data in the dataset and tested against the held-out data. This procedure was repeated until all data-points had been held-out once as the testing data. Because nearly the entire dataset was used for training, and the trained model was close to the real one, we expected the LOOCV error bias to be small. The corresponding *P-*value for the classification for painful HV vs. pain-free HV was calculated from the null distribution obtained from 10,000 random permutation tests by randomly shuffling the labels of samples in the dataset. The *p-*values were calculated as a proportion of the number of permutations generated that were greater than or equal to actual classification accuracy, and the total number of permutations. If none of 10,000 permutations reached the actual accuracy, the *P-*value was *P* < 0.0001.

### Analysis 3: Sequential backward selection and exhaustive search for identifying an optimal feature subset *via* MVPA

The sequential backward selection procedure was initiated from the full set of features; at each iteration we compared the performances of different models built by sequentially removing each of the features from the current set of candidate features. We then excluded the feature whose subtraction resulted in the most increase in classification accuracy from the next iteration the feature. This procedure was repeated until the classification accuracy showed no increase by excluding features.

However, using sequential backward selection could end up with a global optimum rather than a local optimal feature subset. The exhaustive search was also performed to enumerate all possible models for classifying painful HV vs. pain-free HV. The *P*-value for the classification was also generated using permutation test as described in *analysis 2*.

### Analysis 4: Hierarchical clustering analysis for identifying radiographic HV subtype associated with pain in HV

Hierarchical clustering of the 72 HV samples was performed based on homemade MATLAB script using Euclidean distance as distance measure. All other parameters were set to default values. The dendrogram along with silhouette index, Calinski-Harabasz index and Davies-Bouldin index were conducted to determine the optimal number of clusters. Subsequently, the mean of radiographic metrics of each subtype were illustrated in a radar map along with the X-ray of the most representative sample of each subtype (e.g., with the closest distance to the cluster-average). Last, the prevalence of foot pain in each subtype was compared using chi-square test between subtypes.

## Results

### Demographic data

The demographic data and the clinical assessments of all participants are summarized in Table 1. There were no significant inter-group differences with regards to age, sex, or education years (*P* > 0.05).

**Table 1:** Demographic data of painful and pain-free hallux valgus (HV).

	**Painful HV**	**Pain-free HV**	***P*-value**
Age (Years)	58.61 ± 14.67	58.82 ± 14.75	0.86
Sex (female/male)	18/18	18/18	1
Education (Years)	10.64 ± 3.67	11.03 ± 3.54	0.74
VAS score	4.75 ± 2.35	0	0
Duration (Years)	25.67 ± 16.42	25.71 ± 17.68	0.81

*VAS, visual analog scale*.

### Analysis 1: Univariate analyses for exploring radiographic differences between painful and pain-free HV feet

**Figure 1:**
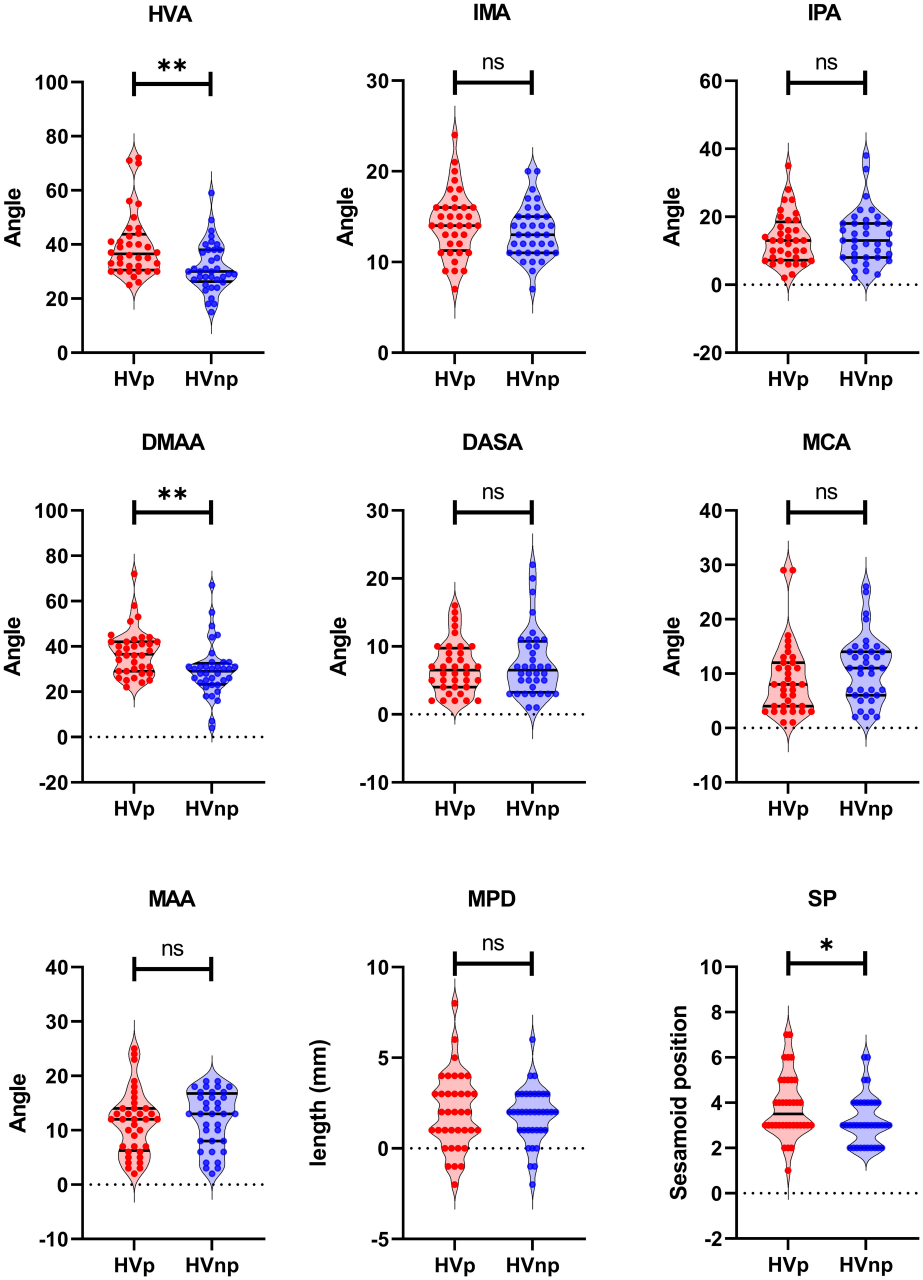
Univariate differences for radiographic metrics between painful and pain-free HV. HVp, Hallux Valgus with Pain; HVnp, Hallux Valgus without Pain; HVA, Hallux Valgus Angle; IMA, Inter-Metatarsal Angle; IPA, Inter-Phalangeal Angle; DMAA, Distal Metatarsal Articular Angle; DASA, Distal Articular Set Angle; MCA, Metatarsal Cuneiform Angle; MAA, Metatarsal Adducent Angle; MPD, Metatarsal Protrusion Distance; SP, Sesamoid Position. **P < 0.005; *P < 0.05. ns means non-significant.

**Figure 2:**
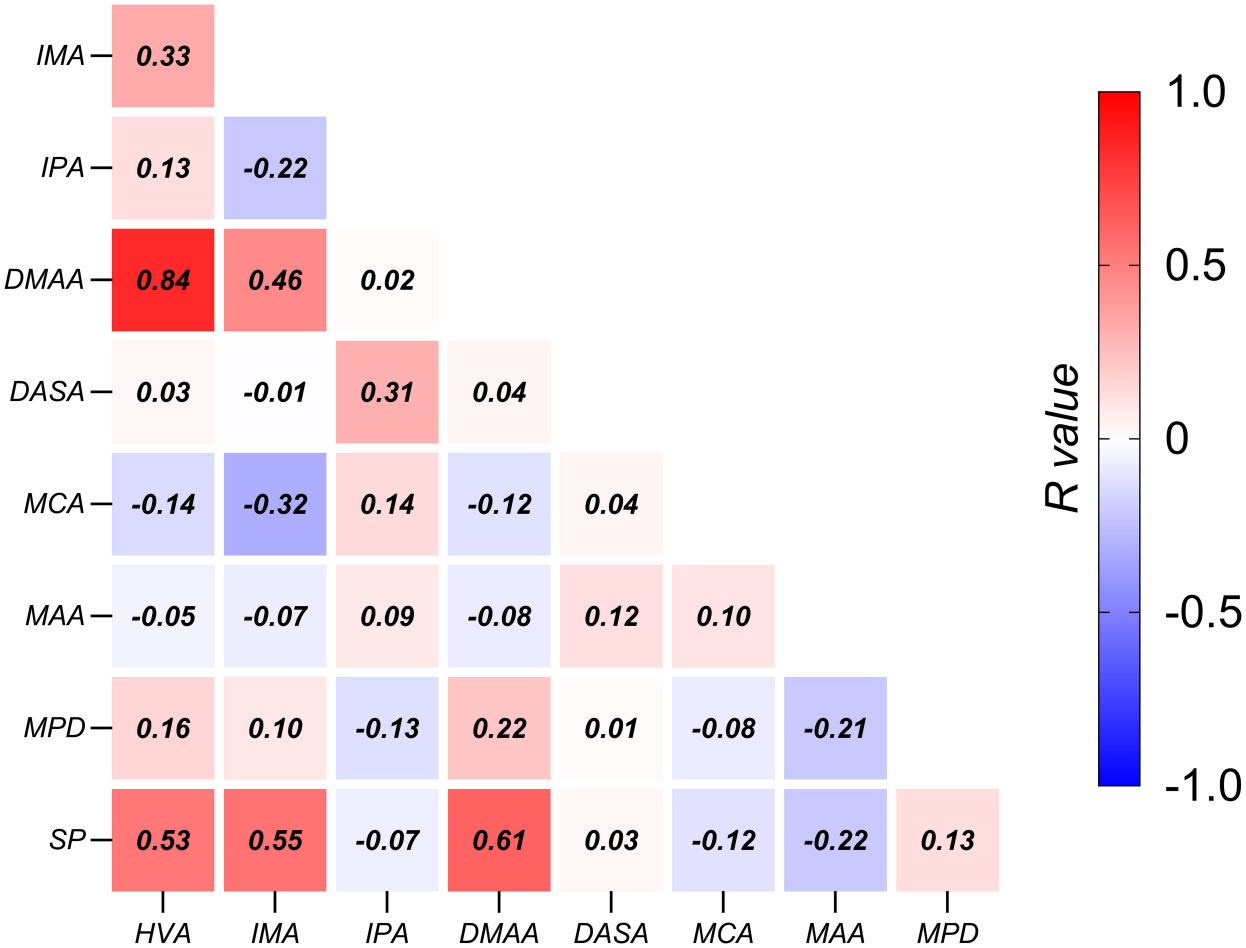
Associations among radiographic metrics in HV. The correlation coefficients are shown in the center of each box. HVA, Hallux Valgus Angle; IMA, Inter-Metatarsal Angle; IPA, Inter-Phalangeal Angle; DMAA, Distal Metatarsal Articular Angle; DASA, Distal Articular Set Angle; MCA, Metatarsal Cuneiform Angle; MAA, Metatarsal Adducent Angle; MPD, Metatarsal Protrusion Distance; SP, Sesamoid Position.

Relative to the pain-free group, the painful group exhibited a significantly higher HVA (*P* = 0.001, Mann-Whitney *U* = 364), DMAA (*P* = 0.001, Mann-Whitney *U* = 369.5) and a higher level of sesamoid subluxation (*P* = 0.04, Mann-Whitney *U* = 477). No significant differences between groups were observed in the following metrics: IMA, IPA, DASA, MCA, MAA, MPD (Figure 1). Significant positive correlations were observed between the following metrics: HVA and DMAA (*R* = 0.84, *P* < 0.001), HVA and IMA (*R* = 0.53, *P* < 0.001), IMA and DMAA (*R* = 0.46, *P* < 0.005), HVA and SP (*R* = 0.53, *P* < 0.001), IMA and SP (*R* = 0.55, *P* < 0.001), and DMAA and SP (*R* = 0.61, *P* < 0.001). Significant negative correlation was observed between IMA and MCA (*R* = −0.32, *P* < 0.05) (Figure 2), however, this negative correlation did not survive the Bonferroni correction.

### Analysis 2: Multi-variate pattern analyses (MVPA) using a support vector machine (SVM) for classifying painful HV feet from pain-free ones

**Figure 3:**
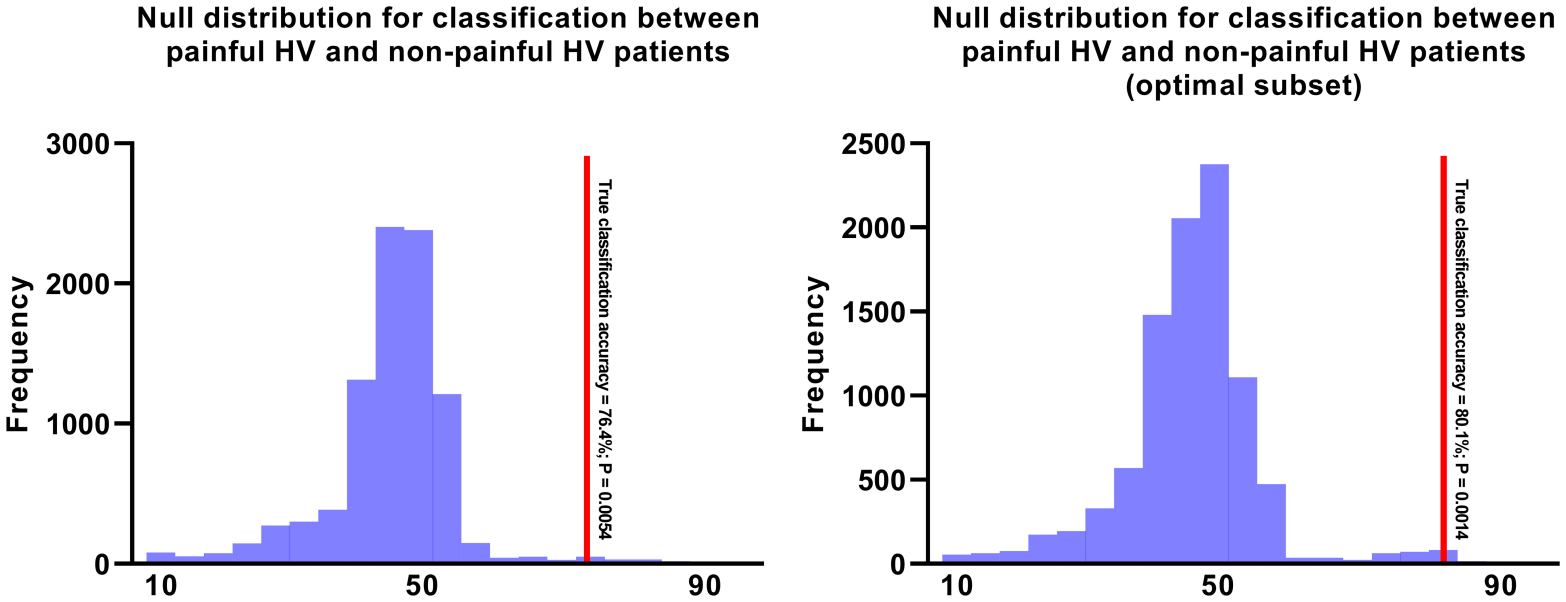
The null distributions for Painful HV vs. Pain-free HV. The left panel illustrates the null distribution of 10,000 permutations for Painful HV vs. Pain-free HV using the entire feature set. The right panel illustrates the null distribution of 10,000 permutations for Painful HV vs. Pain-free HV using the optimal feature set including hallux valgus angle, inter-phalangeal angle, distal metatarsal articular angle, metatarsal adducent angle, metatarsal protrusion distance.

The MVPA was performed to determine whether the pattern of radiographic features could be used to distinguish painful HV feet from pain-free ones. The painful group could be accurately distinguished from the pain-free group using the entire feature set (i.e., all 9 radiographic features) with a 76.4% classification accuracy. The corresponding *P*-value was 0.0054 (Figure 3). This result indicated that a pattern of radiographic features of the foot might accurately predict pain.

### Analysis 3: Sequential backward selection and exhaustive search for identifying an optimal feature subset *via* MVPA

Because *Analysis 2* indicated that the painful HV could be accurately distinguished from pain-free HV using the entire feature set of radiographic measures, the next step was to define an optimal subset of radiographic features that would correlate with HV-related pain. Sequential backward selection procedure was performed, and we found a 5-feature subset with an optimum performance of 80.1% classification accuracy (*P* = 0.0014; Figure 3). Moreover, the exhaustive search also identified the same subset. The 5-feature subset consisted of the following 5 metrics: HVA, IPA, DMAA, MAA and MPD.

### Analysis 4: Hierarchical clustering analysis for identifying radiographic HV subtype associated with pain in HV

**Figure 4:**
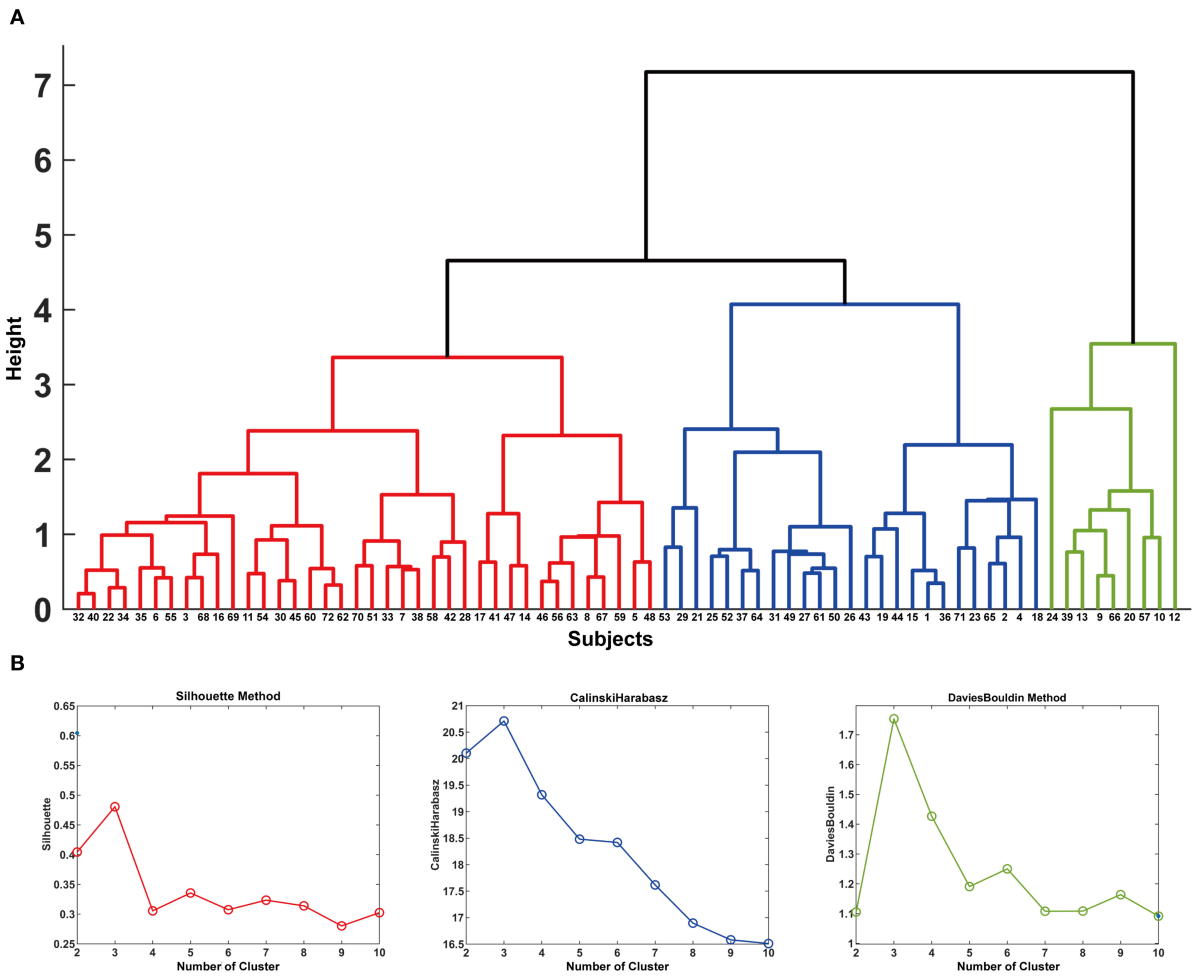
In **(A)**, the dendrogram of the hierarchical clustering is illustrated. The *y*-axis shows the study-specific distances between the clusters as height h. When *h* = 0, each volume forms its own cluster, and *h* = 2 corresponds to splitting the volumes into 13 clusters. The *x*-axis shows the number for each subject. The results of Silhouette index, Calinski-Harabasz index and Davies-Bouldin index analyses are shown in **(B)**.

**Figure 5:**
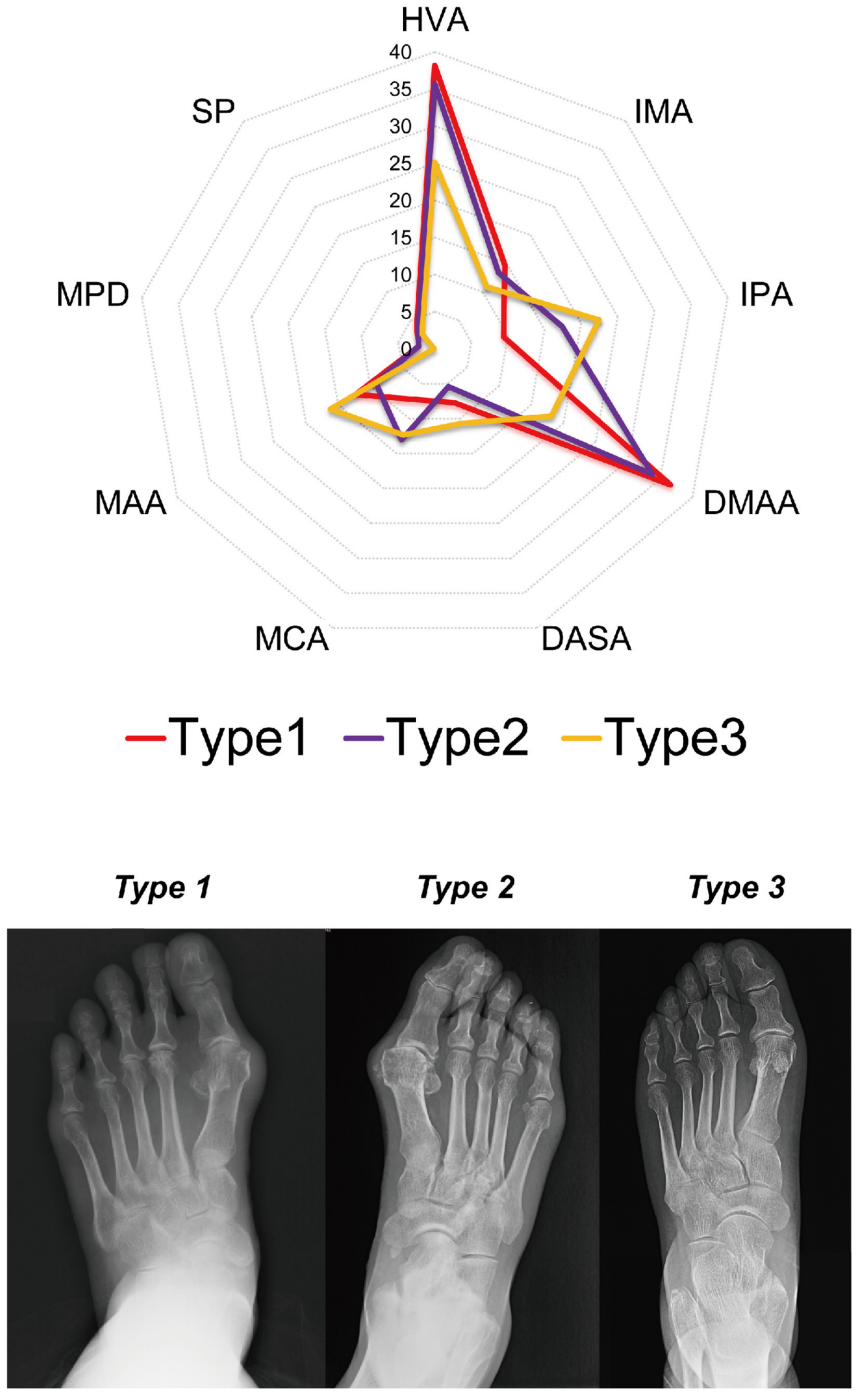
The characteristics of each subtype are illustrated in the radar map and the most representative images of each subtype are illustrated (e.g., with the closest distance to the cluster-average). HVA, Hallux Valgus Angle; IMA, Inter-Metatarsal Angle; IPA, Inter-Phalangeal Angle; DMAA, Distal Metatarsal Articular Angle; DASA, Distal Articular Set Angle; MCA, Metatarsal Cuneiform Angle; MAA, Metatarsal Adducent Angle; MPD, Metatarsal Protrusion Distance; SP, Sesamoid Position.

**Figure 6:**
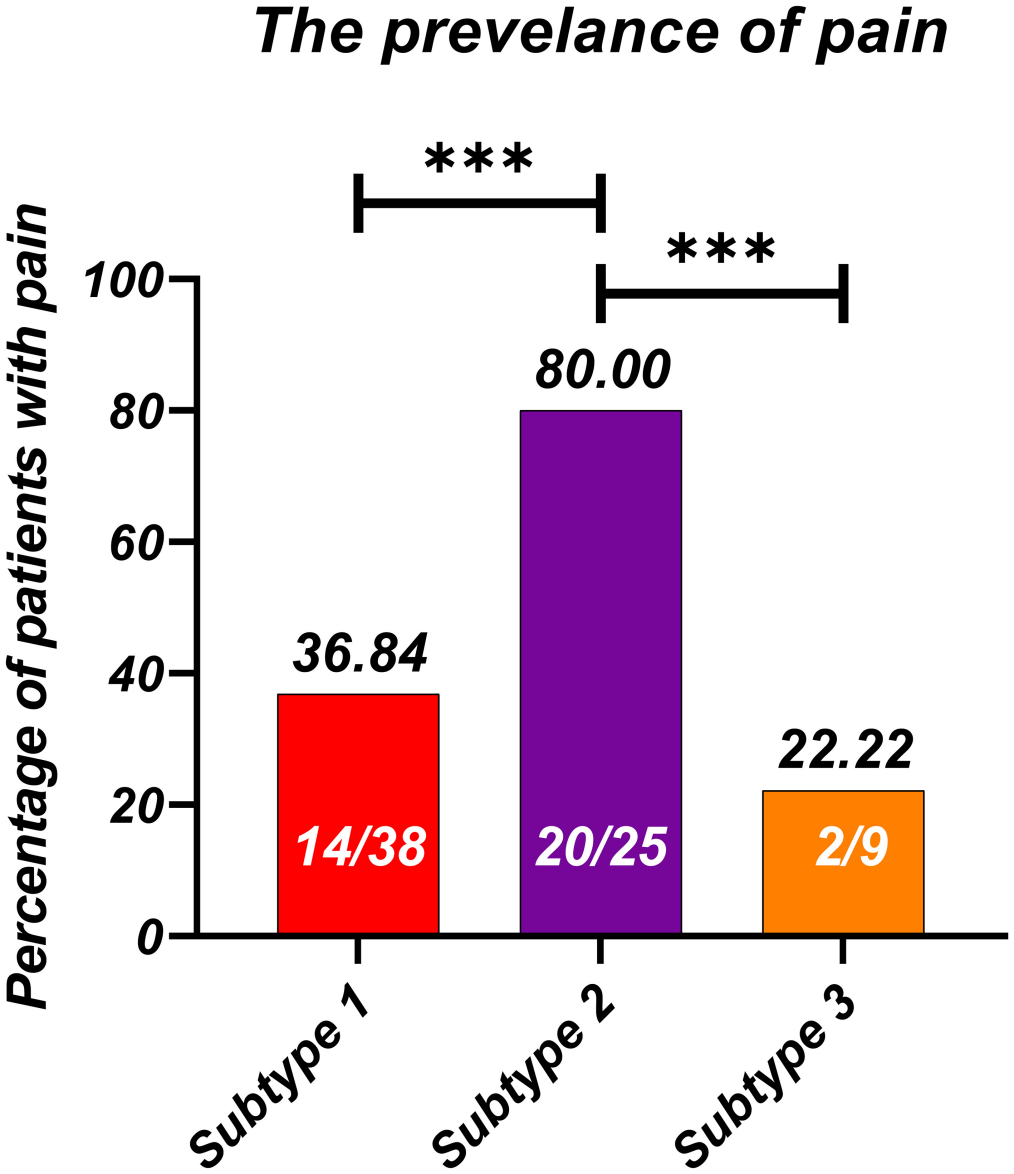
The incidence of foot pain in each radiographic subtype. ***P < 0.001.

The dendrogram showed that the optimal number of clusters was 3 (Figure 4A). The results of Silhouette index, Calinski-Harabasz index and Davies-Bouldin index analyses also showed the optimal number of clusters was 3 (Figure 4B). After hierarchical clustering, 3 subtypes were identified: subtype 1 had the largest sample size (e.g., 38 HV), subtype 3 had the smallest sample size (e.g., 9 HV), and the sample size of subtype 2 was 25. The characteristics of each subtype were illustrated in the radar map and the most representative images of each subtype were also illustrated (e.g., with the closest distance to the cluster-average; Figure 5). Furthermore, the incidence of foot pain was highest in subtype 2 (e.g., 80%; Figure 6).

## Discussion

The three main findings of the present study were: (1) relative to the pain-free group, the painful group exhibited higher HVA, DMAA, and these two metrics were positively correlated; (2) the painful group could be accurately distinguished from the pain-free group using a MVPA. Using sequential backward selection and exhaustive search, an optimal subset was developed that contained 5 radiographic metrics: HVA, IPA, DMAA, MAA and MPD that had optimal performance for classifying painful and pain-free HV; and (3) by applying hierarchical clustering analysis, a radiographic subtype associated with foot pain was also identified.

### Radiographic correlates of pain in HV patients illustrated by univariate analysis

Previous studies had correlated higher HVA with foot pain in HV patients, to which the present study provided further evidence that increased degrees of HV deformity were associated with worse pain in HV. This finding corroborated prior research that had shown pain intensity to be directly correlated with higher degrees of HV deformity. However, whether HV deformity is the major cause of foot pain remains controversial (2, 6, 7, 9, 10). Foot pain in patients with HV has also been associated with patient characteristics such as general health status and occupational physical activity levels rather than severity of hallux deviation (2). Our results partly support with the previous reports that pain intensity is correlated with HV deformity in HV patients. The reasons for contradiction reports were two folds: (1) the inclusion criteria vary from different studies. In our current study, we included HV patients with moderate to severe deformity (i.e., hallux valgus angle above 20°). Several past studies have a relatively wide inclusion criteria that using a HVA above 15° as an inclusion criterion (2, 6, 7). The biomechanical compensatory changes of the foot could partly neutralize the pain and might affect the association between pain intensity and degree of deformity; (2) past studies only explore the univariate association between HV deformity and pain intensity, ignored subtypes which determined by the combination of various angles measured from the HV foot. In our current study, we identified a radiographic painful subtype and two painless subtypes. Interestingly, one of the painless subtypes has a HV angle close to painful subtype, indicating that pain in HV patients is multifactorial.

### The pain of HV is multifactorial, determined by multiple radiographic measurements

In past studies, a large proportion of HV patients with variability of pain intensity remained unexplained. These controversial results were mainly based on univariate analyses, with no attention to associations of other radiographic variables. In the current study, the results of clustering analyses showed that subtypes 1 and 2 exhibited no differences in HVA yet there was significant difference in pain incidence. This finding further supported the idea that pattern information of radiographic variables was crucial in correlation with HV-related pain. The direct comparison of HVA between pain and non-pain participants and liner regression are straight-forward procedures that reveal the association between pain and HVA. However, it only characterizes the quantitative liner association between HVA and pain intensity, which neglects the relationships of HVA and other radiographic measures. Using the MVPA approach and pattern information, the structural morphology of the entire foot was characterized. Recently, studies have shown that HV affected not only the first metatarsophalangeal joint but also the structure of the entire foot including the transverse arch structure (12) and sesamoid platform that led to the biomechanical changes in the foot (11). These findings indicated that the hallux valgus deformity was a multiplanar deformity that affected a wide range of foot structures and could not be adequately evaluated by HVA alone. Our current conclusion that the pattern of radiographic measures could be used to classify painful and pain-free HV also supported this concept. By applying feature selection procedures such as sequential backward selection and exhaustive search, an optimal feature subset which included HVA, IPA, DMAA, MAA and MPD was identified with the best classification performance. Therefore, the pattern of these metrics appeared to have strong correlation to foot pain in patients with HV.

### Clinical significance

In our current study, we found that pain of HV was multifactorial and determined by multiple radiographic measurements. By applying sequential backward selection and exhaustive search, 5 measurements were identified as the optimal feature subset which showed optimal performance classifying painful and pain-free HV feet. It is worth mentioning that the importance of these metrics could not be ranked by our current procedure, and ranking the importance of these metrics would be partial considering our current data failed to include other pain-related factors such as soft tissue of the forefoot, which is also crucial to the pathology of HV. In clinical practice, surgical approaches were designed to correct HVA, DMAA and IMA. By applying distal soft tissue (e.g., McBride procedure) surgery and osteotomies (e.g., Chevron osteotomy, Scarf osteotomy, etc.), these deformities could be significantly corrected (i.e., HVA, IMA) and were effective to alleviate symptoms in HV patients (21). Therefore, from clinical aspect, these metrics would be more of importance than other measurements (3). The deformities measured by IPA, MAA and MPD were corrected only in patients with specific symptoms (e.g., kiss corn, metatarsus adductus and metastatic metatarsalgia) or with residual interphalangeus following metatarsal osteotomy (23). In general, our current results were consistent with clinical experience that the corrective surgery for HV should be holistic rather than only correct the HVA, DMAA and IMA. Our current results provided theoretic basis for this clinical consensus. More importantly, our results suggested that there is a pain-free HV subtype which exhibited similar HVA, DMAA and IMA to painful HV subtype. The differences between these two subtypes were IPA, MCA, indicating that when metatarsal osteotomy was unsatisfactory, other procedures for correcting IPA and MCA [e.g., Akin procedure (23) and Lapidus procedure] would be of potential efficacy for reducing clinical symptoms in HV patients. To our knowledge, the present study is the first conducted using MVPA to identify radiographic correlates of pain in HV patients. Future studies are still needed to validate our results.

## Limitations

Regarding limitations in our current study: (1) we had no external validation set to verify our current results, so multi-center studies with larger sample sizes are needed; (2) we only included the axial images of plane radiographs, future studies are needed to encompass sagittal or even 3-dimensional imaging; (3) our current study is retrospective, lacking assessments of psychological factors which are also considered influential in HV patients with foot pain; (4) our current sample size is relatively small according to the Vapnik-Chervonenkis Dimension of the linear classifier. SVM is well-suited for modeling small samples with powerful predictability, and we also conducted leave-one-out-cross-validation (LOOCV) to include as much data as possible in the training set for model training to minimize the possible effect of small sample size. Still, future study is needed to enlarge the sample size.

## Conclusion

The pain in HV is multifactorial and associated with a radiographic pattern measured by the various angles on plain radiographs. The combination of HVA, IPA, DMAA, MAA and MPD showed the optimal classification performance between painful and pain-free HV.

## Data availability statement

The original contributions presented in the study are included in the article/Supplementary materials, further inquiries can be directed to the corresponding author/s.

## Ethics statement

The studies involving human participants were reviewed and approved by Tianjin Medical Univerisity General Hospital. The patients/participants provided their written informed consent to participate in this study.

## Author contributions

CW designed the study. RZhan, CL, and RZhao analyzed the data and wrote the manuscript. ZL, YZ, SL, and HZ collected the data. XC help analyzed the data. RZhao and CW revised the manuscript. All authors contributed to the article and approved the submitted version.

## Conflict of interest

The authors declare that the research was conducted in the absence of any commercial or financial relationships that could be construed as a potential conflict of interest.

## Publisher's note

All claims expressed in this article are solely those of the authors and do not necessarily represent those of their affiliated organizations, or those of the publisher, the editors and the reviewers. Any product that may be evaluated in this article, or claim that may be made by its manufacturer, is not guaranteed or endorsed by the publisher.
