# Supplementary material for: Identification of radiographic characteristics associated with pain in hallux valgus patients: A preliminary machine learning study
Source: Front Public Health. 2022 Aug 10;10:943026. doi: 10.3389/fpubh.2022.943026 (PMC9399654; doi:10.3389/fpubh.2022.943026)
Supplement: Supplementary file 1 [file Data_Sheet_1.docx]

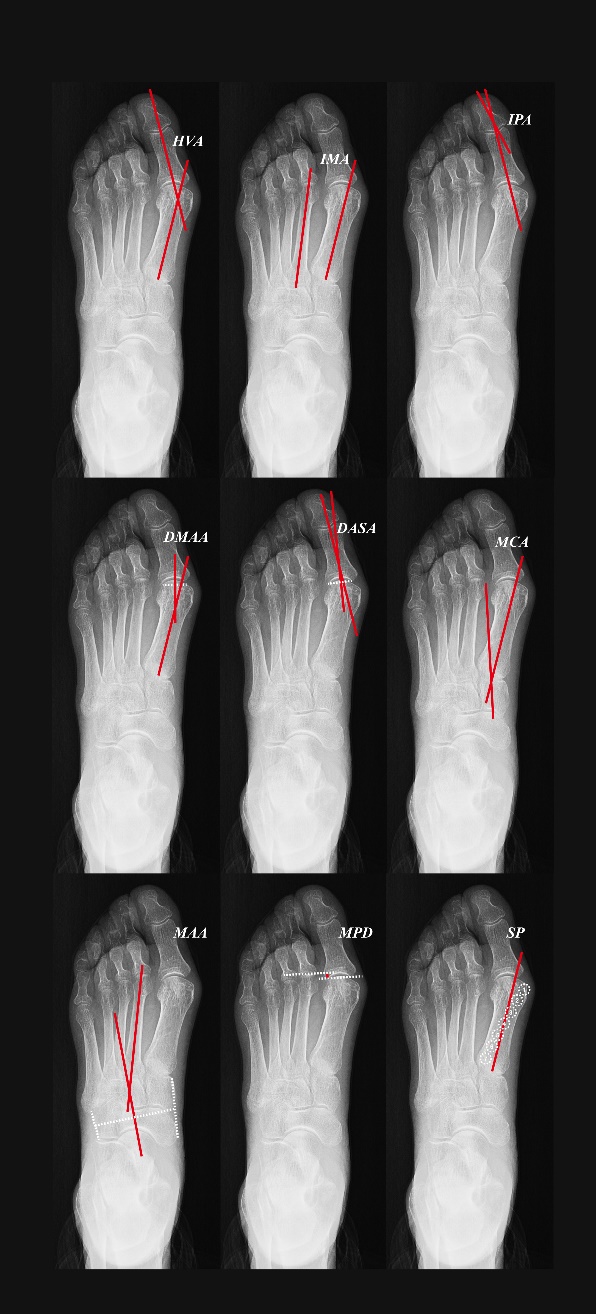


Sup-Fig 1. The radiographic measurements. HVA: Hallux Valgus Angle, IMA: Inter-Metatarsal Angle, IPA: Inter-Phalangeal Angle, DMAA: Distal Metatarsal Articular Angle, DASA: Distal Articular Set Angle, MCA: Metatarsal Cuneiform Angle, MAA: Metatarsal Adducent Angle, MPD: Metatarsal Protrusion Distance, SP: Sesamoid Position


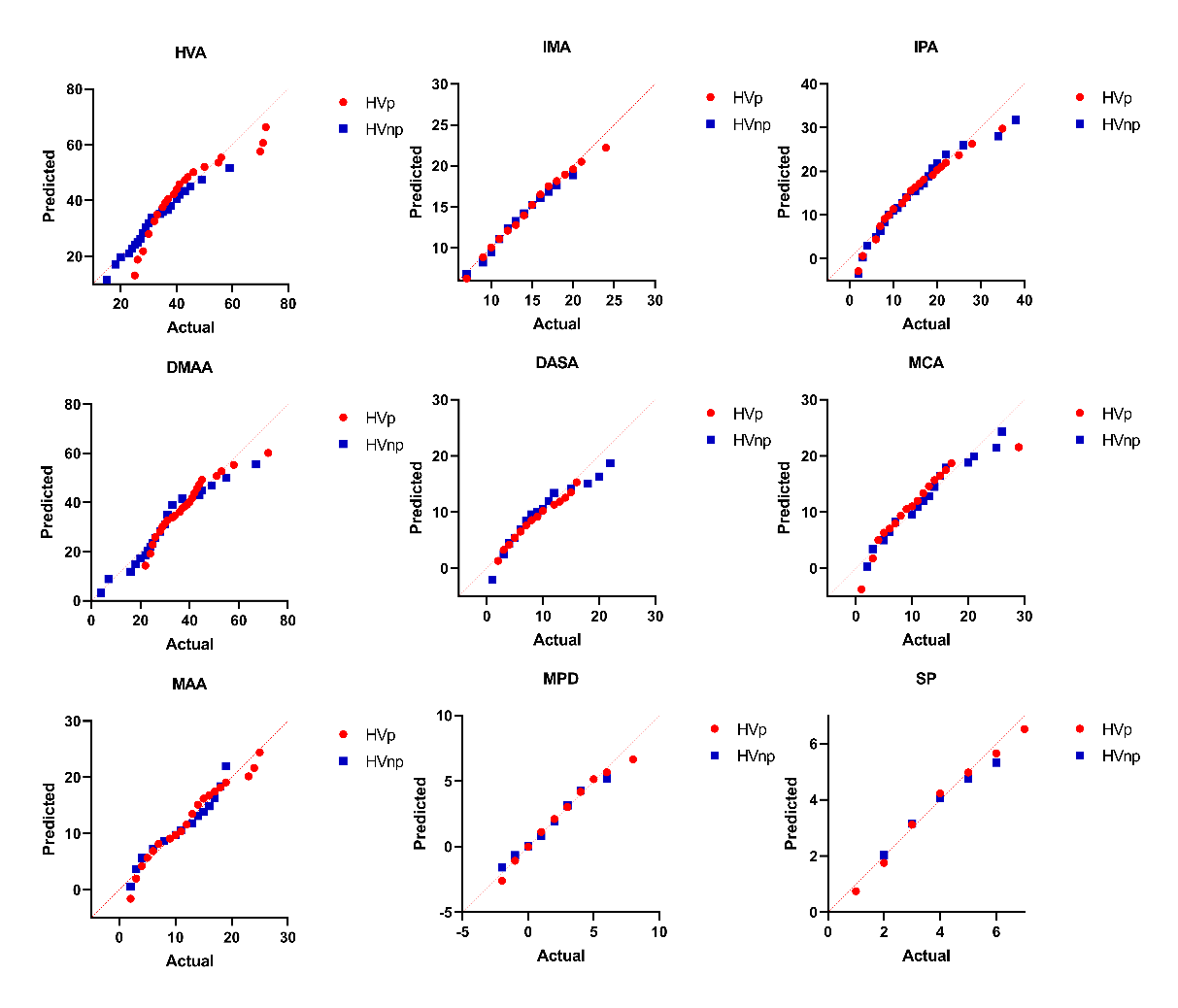


Sup-Fig 2. QQ plots for radiographic data. HVA: Hallux Valgus Angle, IMA: Inter-Metatarsal Angle, IPA: Inter-Phalangeal Angle, DMAA: Distal Metatarsal Articular Angle, DASA: Distal Articular Set Angle, MCA: Metatarsal Cuneiform Angle, MAA: Metatarsal Adducent Angle, MPD: Metatarsal Protrusion Distance, SP: Sesamoid Position
